# Abstract of the Theory of Puerperal Fever; from a Letter Communicated, to the Editors of the American Medical Repository

**Published:** 1799-06

**Authors:** John Brickell

**Affiliations:** Savannah


### *Abſtract of the Theory of Puerperal Fever; from a Letter communicated, to the Editors of the American Medical Repoſitory: By Dr.* John Brickell *of Savannah.*

THE author of this ingenious letter prefaces his new theory of childbed fever, with the following remark:

"During my courſe of anatomical ſtudies, which continued ſeveral years, I had ſome valuable opportunities of diſſecting women who died of puerperal fever; and, upon peruſing treatiſes written expreſſly on this ſubject by the Britiſh phyſicians, *Denman, Leake, Hulme, White,* and *Young,* I readily perceived ſuch a mixture of truth, error, and miſapprehenſion, as convinced me that they had examined this buſineſs too ſlightly, and raſhly made up their opinions from partial and defective premiſes;" &c.

After having, rather ſeverely, animadverted upon ſome particular parts of theſe theories, and furniſhed us with a new hypotheſis relative to the mechaniſm of child-birth, maintained upon ſtatic and mathematical principles *, the author thus proceeds in drawing the *practical inference* from his reaſonings:

"From

---

* The particulars of this theory we propoſe to give in our next.

" From what has been ſaid, it is evident, that the blood is the great inſtrument (cauſe) of danger, from its *exceſs* in the abdomen, and its *defect* in the head; its exceſs producing fatal inflammation and fever, and its defect producing ſyncope and ſpaſms, that ſometimes carry off the patient ſoon after delivery; our great attention, therefore, ought to be directed to the regulation of its motions, ſo as to detach from the abdomen the ſuperabundance, and leſſen its momentum there; and to reſtore to the brain the quantity diverted from it, and which is neceſſary to remove its collapſe, and re-eſtabliſh the libration of the nervous ſyſtem.

" For this purpoſe, a broad bandage or two may be placed under the woman, ready to be properly tightened over a ſmall pillow on the abdomen, as ſoon as delivery is effected; to ſerve as an artificial preſſure and reſtraint on the influx of the blood (inſtead of the uterine preſſure before the birth), and the abdomen ought to be elevated above the horizon, and the head depreſſed, that the velocity of the blood may be checked by its gravity, and its return to the head accelerated by the ſame cauſe. If cordials be given before this, the heart will be ſtimulated to act with more vigour, the abdomen will be more filled with blood, and the head more emptied; and ſo the danger increaſed.

" Should entiritis, peritonitis, epiploitis, or any other inflammation, enſue, I apply the antiphlogiſtic means; but with reſerve, on account of the natural diſcharges. I ſometimes draw blood from the arm; and, in caſes of extreme violence, I have repeated this operation three times in one day, with the happieſt effects.

" I once diſſected a woman who died of a moſt violent puerperal fever. The whole ſurface of the peritonæum, ſtrictly ſo called, as well as the parts of it which give the exterior coats to the inteſtines, uterus, bladder, &c. had, with other marks of high inflammation, a coat of pus, like that obſerved in the corner of an inflamed eye, and as thick as broad cloth.

" As I write for men, I do not find it neceſſary to repeat the particulars to be found in every practical treatiſe: my principal aim being to eſtabliſh a juſt theory, which muſt be prodoctive of a reaſonable practice."

